# Daily torpor in summer- and winter-acclimated hamsters: are thermal biology and balancing energy budgets under a different seasonal physiological control?

**DOI:** 10.1007/s00360-026-01654-3

**Published:** 2026-02-14

**Authors:** Fritz Geiser, Claudia Bieber, Thomas Ruf

**Affiliations:** 1 Research Institute of Wildlife Ecology, Department of Interdisciplinary Life Science, Department of Interdisciplinary Life Sciences, Savoyenstr. 1, Vienna, 1160 Austria; 2 Centre for Behavioural and Physiological Ecology, Zoology, University of New England, Armidale, NSW 2351 Australia

**Keywords:** Daily torpor, Energy conservation, Hibernation, *Phodopus sungorus*, Photoperiod, Season, Survival

## Abstract

Djungarian hamsters (*Phodopus sungorus*), originating from Asian steppes, change fur color from brown in summer to white in winter when they reduce body mass and size of reproductive organs. The species also enters energy-conserving daily torpor, characterized by a substantial temporal reduction of metabolic rate (MR) and body temperature (T_b_). However, spontaneous daily torpor (food ad libitum) is only used by winter-acclimated hamsters. In contrast, short and shallow torpor induced by food restriction may also occur in summer- acclimated hamsters. To better understand the seasonal physiology of torpor, we examined patterns of spontaneous and induced torpor in two groups of hamsters, one was maintained under natural photoperiod in winter (‘winter’ hamsters) the other on a constant long summer photoperiod (‘summer’ hamsters); both were maintained in unheated rooms with an ambient temperature (T_a_) of 9 ± 2 °C during the time of measurements in late winter. Largely white winter hamsters (*n* = 9 of 10), entered spontaneous torpor frequently (51.5% of days), the heaviest individual did not enter torpor. Conversely, none of the brown summer hamsters (*n* = 8) entered spontaneous torpor. Induced torpor at T_a_ 15 °C was observed in both groups (9 of 10 winter hamsters, 2 of 8 summer hamsters), but against predictions, torpor in summer hamsters was on average deeper (minimum T_b_ 19.4 ± 1.1 °C) than in winter hamsters (minimum T_b_ 22.2 ± 1.6 °C). Other variables of torpor were similar although the bout duration tended to be longer in summer hamsters. These data suggest that the seasonal change of physiology of *P. sungorus*, largely caused by photoperiod, does not concern thermal energetics or depth of torpor. These appear to remain similar throughout the year for survival of acute energetic challenges, and this also applies to some hibernators. Rather, seasonality of torpor seems to reflect largely the proclivity of displaying torpor to overcome likely long-term energy challenges that in cold climates are mainly faced in winter.

## Introduction

Many small endothermic mammals and birds use torpor to deal with energetic and other challenges (Dausmann and Warnecke 2016; Nowack et al. 2017). Torpor is characterized by pronounced reductions in metabolic rate (MR) and body temperatures (T_b_) and is highly effective in minimizing energy expenditure and water loss (Boyer and Barnes 1999; Withers et al. 2016). Torpor is widely used by birds and mammals from different climate zones all over the globe (Ruf and Geiser 2015) including several hamster species from the vast Asian steppes (French 2008; Chi et al. 2025).

Hibernating mammals can express multiday torpor bouts lasting up to several weeks with a low torpor MR (TMR) often approximating 5% of basal MR (BMR) of normothermic individuals in thermo-neutrality and a minimum T_b_ of just below 0 to 5 °C (Ruf and Geiser 2015). Many hibernators use torpor predominately, but not exclusively, in the cold season, many rely on stored fat accumulated in autumn for energy supply during hibernation and many do not forage or forage only little in winter (Bieber et al. 2014; Fjelldal et al. 2024; Krivek et al. 2025). In contrast, daily heterotherms, which are restricted to the use of daily torpor lasting for several hours with MR of about 30% of BMR and T_b_ typically around 18 °C, are normally less seasonal and may use torpor throughout the year or even enhance torpor use in summer (Levy et al. 2011; Ruf and Geiser 2015; Geiser 2021). Daily heterotherms do not fatten substantially, some even reduce body mass in autumn and most forage throughout the winter (Geiser 2021).

One apparent exception is the Djungarian hamster (*Phodopus sungorus*), a small Asian hamster with natural populations living in steppes of Kazakhstan and Russia with thermally extreme seasonal climates. This hamster expresses daily torpor exclusively, but shows a strong seasonality of torpor use (Steinlechner et al. 1986). Spontaneous daily torpor (food ad libitum) of captive *P. sungorus* occurs only during the cold months of the year or when hamsters are acclimated to a short winter photoperiod (Heldmaier and Steinlechner 1981; Ruf et al. 1993; Geiser et al. 2013; Cubuk et al. 2016). In summer or when acclimated to a long summer photoperiod, *P. sungorus* does not express spontaneous torpor. This hamster not only changes its use of spontaneous daily torpor with season, but also its body size and sex organs from large in summer to small in winter, its circulation of hormones, its composition of fatty acids in depot fat and muscle, and its pelage colour from brown in summer to largely white in winter (Heldmaier and Steinlechner 1981; Steinlechner et al. 1986; Geiser et al. 2013; Cubuk et al. 2016). Nevertheless, when food is restricted, ‘induced’ torpor may be used even in long summer photoperiod-acclimated *P. sungorus*. However, it was suggested that this type of torpor differs from the spontaneous torpor in winter-acclimated individuals (Diedrich et al. 2015; Cubuk et al. 2016). The induced bouts of torpor in the food restricted summer-acclimated hamsters were short and shallow with T_b_ barely below 30 °C and a TMR that was high at almost 70% of BMR (Diedrich et al. 2015).

To gain a better understanding of the seasonal expression and thermal energetics of spontaneous versus induced torpor in *P. sungorus*, and to examine whether these torpor patterns may be governed by different physiological pathways, we aimed to quantify the occurrence and patterns of spontaneous and induced daily torpor during winter in two groups of hamsters. One group was exposed to a natural short winter photoperiod, whereas the other was exposed to a constant long summer photoperiod under the same thermal conditions.

## Methods

### Animal maintenance

Adult *P. sungorus* born in August/September (i.e. 4–5 months old, > 3 months post-weaning at the beginning of experiments) were used for our study. Two groups were studied. The first group of hamsters (*n* = 10, 5 females, 5 males) were held in a unheated room at the Research Institute of Wildlife Ecology in Vienna under a natural photoperiod from summer (LD ~ 16:8) to winter (LD ~ 10:14) in February when most measurements were performed, and torpor is regularly expressed (Jefimow et al. 2011); these winter-acclimated hamsters are referred to as ‘winter’ hamsters. The second group of hamsters (*n* = 8, 4 females, 4 males) were held under constant long photoperiod (LD 16:8) in a separate unheated holding room and measured at the same time as the ‘winter’ hamsters in winter; these summer-acclimated hamsters are referred to as ‘summer’ hamsters.

As non-reproductive *P. sungorus* are strongly solitary, animals were held individually. Each cage was provided with wood shavings and paper for nest construction. During holding periods hamsters were fed *ad libitum* with standard hamster chow (ssniff^®^HA, ssniff GmbH, Soest, Germany); water was accessible *ad libitum*. Animals were checked daily, and T_a_ was recorded. Measurements were conducted between 1 February and 10 March when T_a_ in the holding rooms was 9 ± 2 °C.

### Body temperature measurements

To measure T_b_ throughout the experiments, all individuals were implanted intraperitoneally with calibrated small temperature data loggers (custom made at the Research Institute of Wildlife Ecology, storage capacity 100.000 temperature readings, accuracy ± 0.1 °C, programmed to record T_b_ at 2-min intervals). Logger weight after coating in Paraffin/Elvax was 1.7 g, which is well below the evidence-based recommended 10% of the body mass for implanted devices in small terrestrial mammals (Rojas et al. 2010). Coated loggers were sterilized before implantation. Surgical anaesthesia was induced by subcutaneous injection of 75 mg/kg Ketamine (Ketamidor^®^ 10%, Richter Pharma Wels, Austria) and 300 µg/kg Medetomidine (Domitor^®^ 0.1%, Orion Corporation, Turku, Finland) and maintained by approximately 1.5% isoflurane in an oxygen stream via a facemask. Preemptive post-surgical analgesia (1 mg/kg Meloxicam) was provided subcutaneously. The animals were placed in dorsal recumbency on a heating pad and the operation field was prepared according to standard surgical procedures and covered by sterile surgical drapes. A midline incision was made, and the abdominal cavity was opened through a ~ 1 cm incision along the *linea alba*. Post implantation, peritoneum and abdominal muscles were sutured using synthetic absorbable surgical suture material USP 4/0 (Surgicryl PGA, SMI AG, Hünningen, Belgium). The skin was sutured separately using the same synthetic absorbable surgical suture material. During the entire procedure, vital parameters (respiration rate, peripheral haemoglobin oxygen saturation as measured by pulse oximetry, pO_2_, heart rate) were monitored. After implantation, animals were placed into their cages and the healing process was checked daily. Animals were allowed to recover from surgery at near thermo-neutral T_a_ (23 to 25 °C) for 11 days. Implantations were carried out on 20 January and loggers were removed on 25 March.

### Metabolic rate measurements

Metabolic rates of hamsters were measured as the rate of oxygen consumption using open flow respirometry from 27 February to 10 March. The analyser (a Servomex paramagnetic oxygen analyser, Servopro 4100, Servomex, Crowborogh, UK) was calibrated before measurements commenced and once during the measurement period. Animals were placed into 750 ml Perspex respirometry tubes that allowed free movement and were placed within a temperature-controlled cabinet (TPK600, Feutron, Langenwetzendorf, Germany). Respirometry tubes were sealed on either end by a rubber stopper containing inlets for air on one end and outlets on the other, and the inlet for shielded thermocouple probes to measure the T_a_ in the respirometry tubes. During respirometry measurements, a thin layer of wood shavings was provided on the respirometer floor for absorption of urine and feces. To induce torpor, food and water were not provided for 18–22 h; all animals were well and alert at the end of the measurements. The respirometry tubes were 25 cm long, 12 cm of which was covered by a cardboard tube to provide a refuge for the animals. The flow rate, measured with mass-flowmeters (FMA 3100, Omega Engineering, Stamford, CT, USA), through the respirometry chamber was about 800 ml/minute (i.e. time to 99% equilibrium was ~ 4 min). Four individual animal channels and one control channel (outside air) were measured in sequence for 1 min each, therefore a reading of each animal was taken every 5 min. Channels were switched via solenoid valves and the washout from the tubing to the analyser was achieved within 10 s. The T_b_ was measured throughout these measurements as outlined above using the implanted temperature loggers. Animals were measured overnight (from late afternoon to ~ 14:00 h) to induce torpor at T_a_ 15 ± 1 °C and were observed through a window during the daytime when measurements were conducted. Each animal was measured 1 to 3 times and was rested with free access to food and water for > 4 days between measurements.

### Calculations, definitions and statistics

Metabolic rates were calculated over at least 15 min during the longest and deepest undisturbed torpor bout when values were minimal and stable and calculated according to Withers (1977). The corresponding T_b_ and T_a_ were averaged over the same time period. The torpor threshold was defined as T_b_ <30.0 °C (i.e. a fall of T_b_ by > 5 °C below the normothermic, resting T_b_ of about 35° at T_a_ 15 °C; Ruf and Geiser 2015; Geiser et al. 2016). If more than one measurement was available for individuals, these were averaged, and analyses were conducted on these averages. Two-tailed t-tests were used to determine potential differences between means. Mann-Whitney U-tests were used to analyze non-parametric measures such as torpor occurrence. Numeric values are expressed as means with SD for ‘n’ the number of individuals measured.

## Results

The external appearance and body mass of the two groups of hamsters differed substantially. Winter hamsters were largely white and their body mass at the beginning of metabolic measurements, was 27.3 ± 2.7 g. In contrast, summer hamsters were brown, and their body mass of 38.8 ± 7.0 g was significantly higher (t = 4.5; *p* < 0.001) than that of winter hamsters.

The normothermic T_b_ of hamsters measured in their holding rooms and during metabolic measurements showed strong daily fluctuations. In both groups of hamsters, normothermic T_b_ fluctuated between about 34 and 38 °C.

**Fig. 1:**
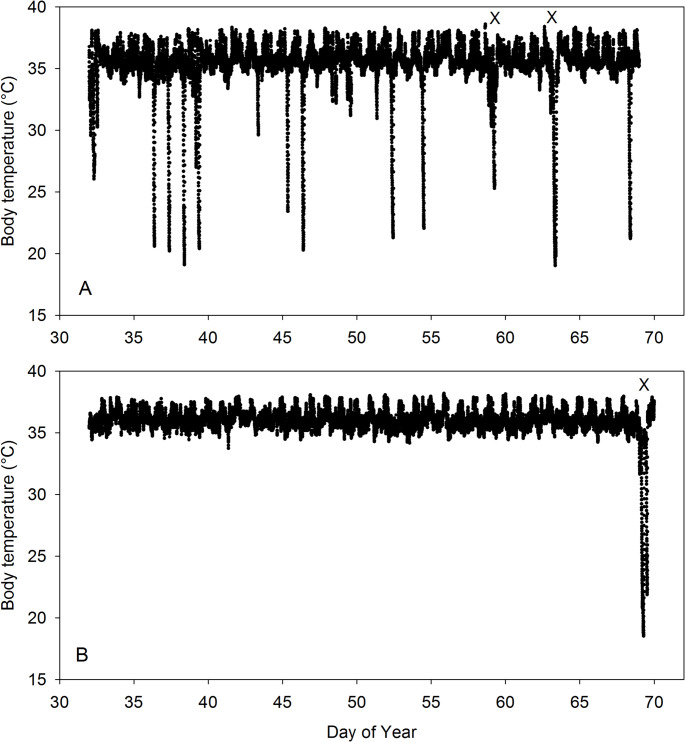
Body temperature (T_b_) of two hamsters measured from 1 February (11 days post-surgery) to 10 March. **A** (Top) shows a white winter hamster that displayed spontaneous torpor (food ad libitum) frequently, as well as induced torpor (X, food withheld). **B** (Bottom) shows a brown summer hamster that never displayed spontaneous torpor but did enter induced torpor (X, food withheld)

Spontaneous torpor was frequently observed in winter hamsters from 1 February to 10 March, and the average number of torpor bouts expressed in 9 individuals was *n* = 19.6 ± 12.5 (Fig. 1; Table 1). One winter hamster, the heaviest male weighing 38 g, did not enter spontaneous torpor. None of the summer hamsters displayed spontaneous torpor throughout the same time-period and torpor occurrences differed significantly between the groups (Fig. 1; Table 1).

**Table 1:** Variables of torpor and body mass measured in winter and summer-acclimated hamsters *P. sungorus* at T_a_ 15 °C for induced torpor, and at T_a_ 9 °C for the number of spontaneous bouts

Body mass (g)	min T_b_ (°C)	min T_b_-T_a_ (°C)	Torpor MR (ml O_2_ g^-1^h^-1^)	Induced bouts (occurrence %)	Longest induced bout (min)	Spontaneous bouts (n)
W: 27.3 ± 2.7	22.2 ± 1.6	7.0 ± 1.8	0.78 ± 0.26	83 ± 36	152.4 ± 79.3	19.6 ± 12.5
S: 38.8 ± 7.0	19.4 ± 1.1	4.7 ± 1.4	0.88 ± 0.29	25 ± 46	237.0 ± 4.0	0
*p* < 0.001	*p* < 0.05	ns	ns	*p* < 0.05	ns	*p* < 0.01
*t* = 4.5	*t* = 2.3			*z*-score = 2.3		*z*-score = 3.2

*W* Winter, *S* Summer, minimum T_b_ and T_b_–T_a_ is min T_b_ and min T_b_–T_a_

**Fig. 2:**
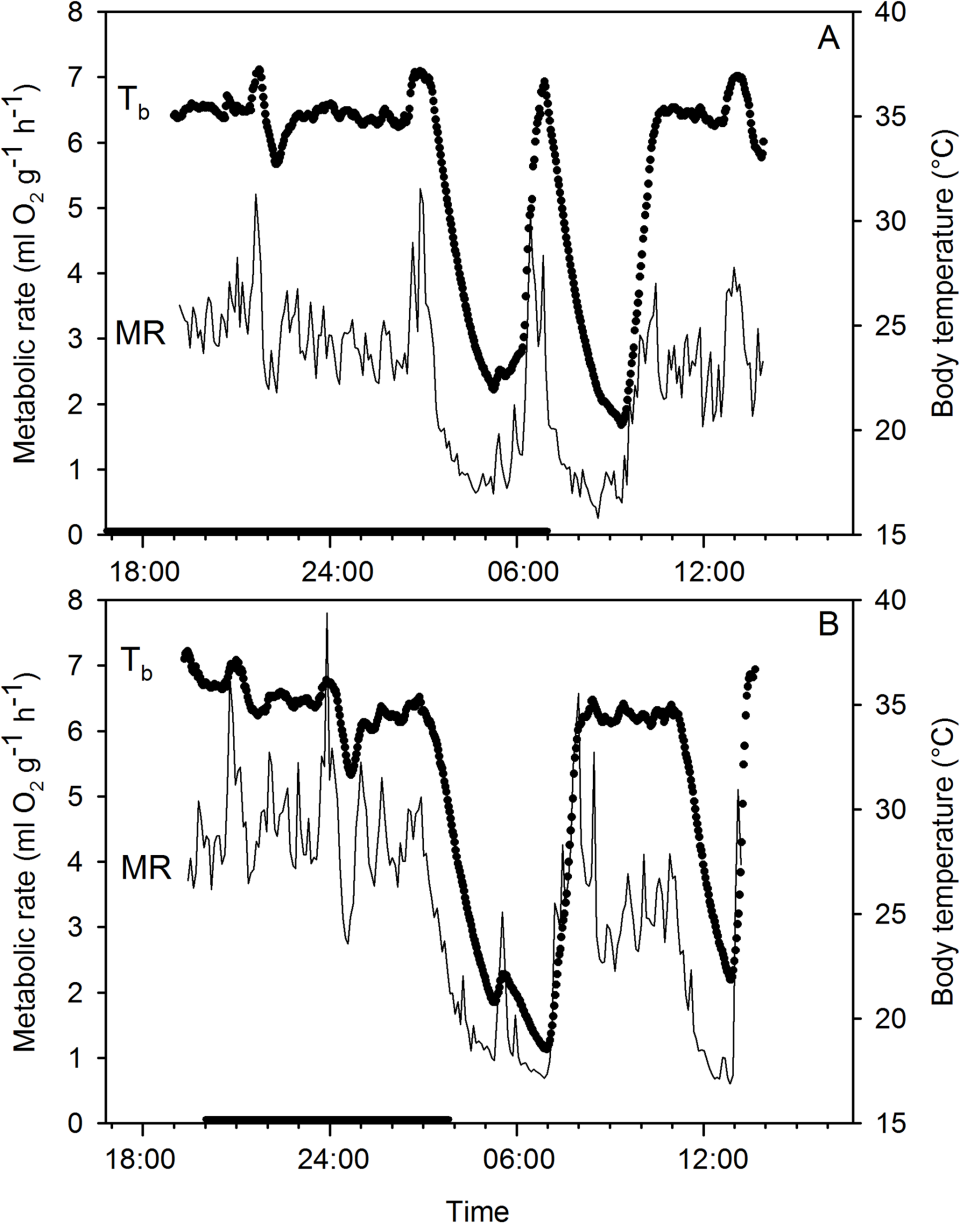
Body temperature (T_b_) and metabolic rate (MR) measured as rate of oxygen consumption in *P. sungorus* as a function of time during overnight measurements. (**A** top) White winter hamster, (**B** bottom) brown summer hamster. No food or water were provided to induce torpor. The black bar on the x-axis indicates night

Induced torpor was observed in both winter and summer hamsters (Figs. 1 and 2). However, 9 of 10 winter hamsters displayed induced torpor, whereas only 2 of 8 summer hamsters did so (Table 1). The same male winter hamster that did not display spontaneous torpor also did not display induced torpor although it was measured twice overnight. The summer hamsters displaying induced torpor were both females and were the lightest individuals of the group (30.9 and 33.3 g). In both groups of hamsters, torpor entry was characterized by a rapid fall of MR with a parallel or, because of thermal inertia, a slightly delayed fall in the T_b_ (Fig. 2). Induced torpor entry required about 2–4 h and most hamsters rewarmed soon after the minima of MR and T_b_ were reached. Both hamsters shown entered a second bout of torpor after rewarming to normothermic T_b_ was completed and a normothermic period. Both summer hamsters and 7 of 9 winter hamsters displayed two bouts of torpor. In the winter hamsters a single torpor bout was observed on 6 occasions, a dual bout on 7 occasions and a triple bout on 2 occasions.

Unexpectedly, physiological variables of induced torpor in the summer hamsters were either more pronounced or like those of the winter hamsters (Table 1). The mean minimum T_b_ was significantly lower in the summer than in the winter hamsters (*p* < 0.05, t = 2.3; Table 1). The TMR was 41% of BMR in winter hamsters (BMR = 1.91 ml O_2_ g^−1^h^− 1^, Ruf and Geiser 2015), similar to the 46% in the summer hamsters and the TMR was statistically indistinguishable between the groups (Table 1). Although the T_b_-T_a_ differential and duration of torpor bouts were statistically indistinguishable because of the small sample size for summer hamsters, the mean bout duration in the summer hamsters was about 1.5-fold of that in winter hamsters and the T_b_-T_a_ differential in the summer hamsters were somewhat lower than in winter hamsters (Table 1).

## Discussion

We provide new information demonstrating that daily torpor induced by short-term food withdrawal in summer-acclimated *Phodopus sungorus* can be deeper or similar to that in winter hamsters, although occurrence of torpor was rare. Our study confirms that photoperiod has a strong effect on *P. sungorus* as long photoperiod summer-acclimated hamsters did not display spontaneous torpor when food was available, whereas short photoperiod winter hamsters frequently did so.

Comparatively, our observations on thermal energetics during torpor in both winter and summer-acclimated hamsters were like those reported on winter hamsters previously. The torpor patterns of induced torpor in hamsters observed here were somewhat unusual because hamsters often expressed more than one torpor bout/day, but it is known that multiple bouts/day may be used in the species especially when food is reduced (Steinlechner et al. 1986; Diedrich et al. 2015), as in our measurements. Regarding physiological variables during steady-state torpor of both groups of hamsters studied here, the minimum T_b_ of about 19–22 °C was as measured in winter-acclimated hamsters under similar thermal conditions (Heldmaier and Steinlechner 1981; Diedrich et al. 2015). Somewhat surprisingly, the mean minimum TMR of the summer hamsters measured here was only about half that of most winter hamsters measured by Heldmaier and Steinlechner (1981), but like more recent measurements on winter hamsters (Diedrich et al. 2015; Ruf and Geiser 2015).

So why were patterns of torpor in our summer hamsters more pronounced than in previous studies? In previous investigations, food restrictions by 30 to 60% of the daily intake of hamsters resulted in either no torpor or expression of short and shallow bouts of torpor in summer hamsters (Steinlechner et al. 1986; Dietrich et al. 2015). In contrast, in our study when no food was provided overnight and T_a_ was slightly lower (T_a_ 15 °C vs. T_a_ 18 °C in Diedrich et al. 2015), rather deep and long bouts of torpor were observed in the lightest female summer hamsters. This shows that the summer hamsters are physiologically capable of expressing deep and long torpor when the animal perceives an acute signal of an energy emergency such as lack of food by withdrawal in captivity. For other species this may occur in the wild during fires, storms, droughts or floods when use of torpor is energetically required for survival (Nowack et al. 2017), or for avoidance of predators (Bieber and Ruf 2009; Barratt et al. 2025). Of course, torpor use will be more urgent in light individuals as observed in our study. Spontaneous daily torpor on the other hand is for balancing energy supply and demand in winter when energy is likely limited, as signaled by a short photoperiod. In *P. sungorus* this should typically not be often required in summer when days are long and food typically is abundant. However, even if the summer hamsters are reluctant to express torpor, they have the functional capability to enter and arouse from torpor. Consequently, the strong seasonal response that is often reported for *P. sungorus* and used to examine the likely underlying physiological reasons for the seasonal torpor expression (Cubuk et al. 2016; Jastroch et al. 2016), seems to reflect a seasonal change in proclivity to enter torpor, but apparently not a seasonal change that represents its thermo-energetic capabilities expressed as the depth of torpor used by the animal.

Few studies have compared physiological variables of daily heterotherms between summer and winter, because typically in summer no torpor was observed or examined. However, when summer and winter torpor have been compared the outcomes were not always as expected. In Australian blossom bats (*Syconycteris australis*) and spiny mice (*Acomys russatus*) from Middle eastern deserts, daily torpor is used throughout the year and is more pronounced and longer in summer than in winter to deal with low nectar and water availability (Coburn and Geiser 1998; Levy et al. 2011). In marsupial dunnarts (*Sminthopsis* spp.) from Australian deserts, daily torpor also occurs in summer. Both spontaneous as well as induced torpor bouts were observed and the induced bouts in summer were not quite as deep as in winter with a higher minimum T_b_ (by 2–3 °C) and higher TMR in summer (Geiser and Baudinette 1987). Importantly, all these species maintained a high thermal tolerance and could lower their T_b_ substantially in summer. In North American deermice (*Peromyscus maniculatus*) individuals captured in summer and measured in outdoor cages did not enter spontaneous torpor, but despite much higher summer T_a_s showed similar patterns of torpor and torpor variables as those in winter when food was partially restricted (Tannenbaum and Pivorun 1989), comparable to our study. The use of induced torpor and retained thermal tolerance in summer deermice was interpreted as a short-term survival mechanism available throughout the year (Tannenbaum and Pivorun 1989).

In seasonally hibernating species the thermal tolerance also can be similar in summer and winter. This is well known from classical studies in which summer active European ground squirrels (*Spermophilus citellus*), hamsters (*Cricetus cricetus*) and hedgehogs (*Erinaceus europaeus*) were experimentally cooled in an ice bath (Horvath 1881). The hibernators were compared with homeothermic rabbits and dogs, which when cooled died at a hypothermic T_b_ of about 19 °C (Horvath 1881). In contrast, the minimum hypothermic T_b_ the three hibernating species survived in summer ranged from 1.2 to 2.2 °C (Horvath 1881), similar to the minimum T_b_ of these species during hibernation in winter (Ruf and Geiser 2015; Hoelzl et al. 2015; Geiser 2021). In hibernators with a less pronounced seasonal cycle like marsupials, bats and dormice, deep torpor can be used in summer and both the minimum T_b_ and TMR may be indistinguishable from those in winter (Stawski and Geiser 2011; Geiser 2024). Of course, if measured in outside aviaries or in the wild, T_b_ is likely higher and torpor bouts shorter in summer because of the higher T_a_ (Bieber and Ruf 2009). More recent biochemical studies also show limited seasonal functional changes in hibernators. Antioxidant enzymes in 13-lined ground squirrels (*Ictidomys tridecemlineatus*), show little change between summer active and winter hibernating individuals (Page et al. 2009). Overall, these data do not support the interpretation that the perceived summer and winter phenotypes of seasonal hibernators (Williams et al. 2005) as quantified by seasonal cDNA analyses, drive seasonally different thermal energetics, the very variables that typically are used to describe such seasonal differences in biology. As this does not appear to be the case the cDNA modifications must reflect some other seasonal change. Perhaps they are related to metabolizing lipids, reduction of metabolizing toxic compounds and urea, and a reduced electron transport in the brain (Williams et al. 2005). As the authors point out, the seasonal changes of cDNA were modest in both golden-mantled (*Urocitellus lateralis*) and 13-lined ground squirrels (*I. tridecemlineatus*) (Williams et al. 2005), and there appears to be no support for the view of a profound phenotypic seasonal transition at least not for thermal energetics and depth of torpor.

Clearly our findings have implications for the seasonality of torpor. But they also have consequences for the ever-shrinking number of homeothermic mammals as currently perceived and increasing number of recognised heterothermic species, at least for small species. Supposed homeotherms such as aardvarks, large fruit bats, bush rats and voles have recently been discovered to use torpor, but like summer torpor this had been overlooked. Although these species have been described or viewed as homeotherms for decades, especially when measured in the field, all expressed torpor apparently to deal with energy emergencies. Aardvarks (*Orycteropus afer*) are large (~ 35 kg) burrowing Afrotherian mammals. Aardwarks usually appear to be homeothermic, but in the winter following a summer drought, animals entered bouts of torpor and reduced T_b_ as low as 24.7 °C (Weyer et al. 2020). Similarly, grey-headed fruit bats (*Pteropus poliocephalus*, ~ 800 g) have been described in several laboratory studies as being homeothermic, but in the field during wet, cool and windy weather, bats entered torpor and reduced T_b_ to a minimum of 27 °C (Turbill et al. 2024). Bush rats (*Rattus fuscipes*, ~ 100 g) were also described as homeotherms in several previous studies on thermal biology, but in a field study one individual reduced T_b_ to 24 °C and rewarmed endogenously (Nowack and Turbill 2022). Another perceived homeothermic group are Microtine voles, but a low T_b_ of 26 °C has been observed in captive *Microtus lusitanicus* (17 g) captured in the wild (Monarca et al. 2019). These data suggest that rare use of torpor is not only seen in tropical and subtropical species (Nowack et al. 2023) but also applies to species living in temperate or cold regions during energetic emergencies including during summer.

Thus, in summary, summer homeothermy in heterotherms appear to reflect the proclivity to express torpor rather than a seasonal change in thermal tolerance. Regarding the widely perceived strict mammalian homeothermy, this may be a characteristic that applies only to a minority of species.
